# 4-Oxo-2-phenylchroman-6-yl propionate

**DOI:** 10.1107/S1600536810012298

**Published:** 2010-05-22

**Authors:** Edyta Kostrzewa-Susłow, Agata Białońska, Tomasz Janeczko

**Affiliations:** a Department of Chemistry, Wrocław University of Environmental and Life Sciences, 25. Norwida, 50-375 Wrocław, Poland; b Faculty of Chemistry, University of Wrocław, 14. F. Joliot-Curie, 50-383 Wrocław, Poland

## Abstract

In the structure of the title compound, C_18_H_16_O_4_, both the *S* and *R* enanti­omers appear to occupy in a random way four symmetry-equivalent sites of the unit cell in an approximately 4:1/1:4 ratio. The chiral C atom of the pyrone ring together with the phenyl ring bonded to this atom are disordered over two positions, the occupancy factor of the major component being 0.809 (5). Adjacent molecules are linked by weak C—H⋯O hydrogen bonds.

## Related literature

For background to flavonoids and their properties, see: Harborne & Baxter (1999 ▶); Harborne & Williams (2000 ▶); Di Carlo *et al.*,(1999 ▶); Rice-Evans (2004 ▶); Wang (2000 ▶); Halliwell (1996 ▶); Rice-Evans *et al.* (1996 ▶); Kostrzewa-Susłow *et al.* (2008 ▶). For related structures, see: Shoja *et al.* (1998 ▶); Białońska *et al.* (2007 ▶).
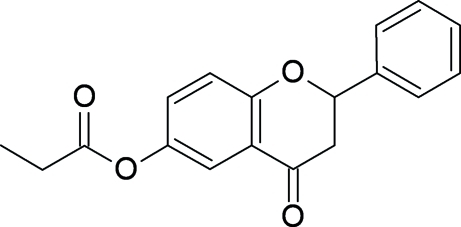


## Experimental

### 

#### Crystal data

C_18_H_16_O_4_
*M* *_r_* = 296.31
Monoclinic, 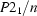

*a* = 7.863 (2) Å
*b* = 17.876 (4) Å
*c* = 10.731 (2) Å
β = 101.28 (3)°
*V* = 1479.2 (6) Å^3^
*Z* = 4
Mo *K*α radiation
μ = 0.09 mm^−1^
*T* = 100 K
0.32 × 0.15 × 0.09 mm

#### Data collection

Kuma KM4 CCD diffractometer
23501 measured reflections
5512 independent reflections
1906 reflections with *I* > 2σ(*I*)
*R*_int_ = 0.126

#### Refinement

*R*[*F* ^2^ > 2σ(*F* ^2^)] = 0.063
*wR*(*F* ^2^) = 0.140
*S* = 0.86
5512 reflections
263 parameters
186 restraints
H-atom parameters constrained
Δρ_max_ = 0.26 e Å^−3^
Δρ_min_ = −0.20 e Å^−3^

### 

Data collection: *CrysAlis CCD*, (Oxford Diffraction, 2009 ▶); cell refinement: *CrysAlis RED* (Oxford Diffraction, 2009 ▶); data reduction: *CrysAlis RED*; program(s) used to solve structure: *SHELXS97* (Sheldrick, 2008 ▶); program(s) used to refine structure: *SHELXL97* (Sheldrick, 2008 ▶); molecular graphics: *XP* (Bruker, 1999 ▶); software used to prepare material for publication: *SHELXTL* (Sheldrick, 2008 ▶).

## Supplementary Material

Crystal structure: contains datablocks global, I. DOI: 10.1107/S1600536810012298/hg2666sup1.cif

Structure factors: contains datablocks I. DOI: 10.1107/S1600536810012298/hg2666Isup2.hkl

Additional supplementary materials:  crystallographic information; 3D view; checkCIF report

## Figures and Tables

**Table 1:** Hydrogen-bond geometry (Å, °)

*D*—H⋯*A*	*D*—H	H⋯*A*	*D*⋯*A*	*D*—H⋯*A*
C2—H2⋯O18^i^	1.00	2.37	3.145 (3)	133

Symmetry code: (i) 
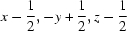
.

**Fig. 1.**
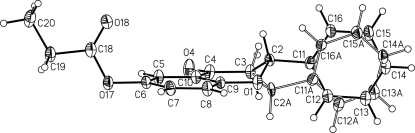
Structure of 6-propionoxyflavanone. Disordered part with occupancy factor equal to 0.2 is marked by open line.

**Fig. 2.**
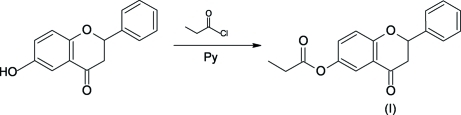
The title compound was obtained during esterification of 6-hydroksyflavanone using propionyl chloride.

## supplementary crystallographic information

### Comment

Flavonoids, which are the subject of our research, are biologically active substances naturally occuring in plants. The colour of flowers and leaves and its intensity is correlated with their presence. Due to the strong UV absorption, flavonoids play protective role in plants. They are also nectar indicators. Flavonoids protect plants from pathogens, act as inhibitors of auxins transport and also initiate formation of root nodules in papilionaceous plants [Harborne & Baxter, 1999; Harborne & Williams, 2000].

So far, flavonoids have not been found in organisms of animals and humans, however worldwide research proved wide range of valuable biological activities of these compounds. These include antiallergic, antiatherogenic, antidiabetic, antidiarrheic, antiinflammatory, antihepatotoxic and anticancerogenic properties [Di Carlo *et al.*, 1999; Rice-Evans, 2004; Wang, 2000]. The wide spectrum of their pharmacological activities depends on chemical structures. Especially important is presence of carbonyl group, as well as presence, number and location of hydroxyl groups. For example, the presence of hydroxyl groups in the B ring is the main factor determining antioxidant activity of flavonoids [Halliwell, 1996; Rice-Evans *et al.*, 1996].

Transformation of flavonoids by means of microorganisms is a way of modification of their structure, as well as a helpful tool for elucidation of their metabolism in mammals [Kostrzewa-Susłow *et al.*, 2008].

The crystal structure of 6-propionoxyflavanone, together with numbering scheme employed, is presented in Fig. 1. In the present analysis, atoms at position 2 in the pyrone ring [C2 and H2 (major component) and C2A and H2A (minor component)] and phenyl ring [C11—C16 (major component) and C11A—C16A (minor component)] are clearly resolved. The C11—C16 (or C11A—C16A) phenyl ring is oriented almost perpendicular to the plane of the C5—C10 arene ring. The angle between the plane of the C11—C16 (C11A—C16A) ring and the plane of the C5—C10 ring is equal to 79.79 (12) ° (89.8 (5) °). The angle between the plane of carboxylate group and the plane of the C5—C10 ring is equal to 75.62 (8) °. The O1, C3, C4 O17 atoms are situated approximately in the plane of the C5—C10 arene ring (maximum deviation is equal to 0.040 (3) Å for O1). While deviation of the C18 and C2 atoms from the plane formed by the C5—C10 arene ring are equal to 1.140 (4) and 0.676 (4) Å, respectively, deviation of the C2A atom from the plane is equal to -0.403 (13) Å. Thus, two enantiomers revealing various conformations occupy equivalent sites, however somewhat randomly, not systematically, arranged in the unit cell. The ratio of the two enantiomers (*R*:*S*) in an asymmetric part of the unit cell is approximately equal to 0.8:0.2, which gives a 4:1/1:4 ratio in the crystal structure overall.

### Experimental

The title compound was obtained during esterification of 6-hydroksyflavanone using propionyl chloride (Fig.2). Crystals of 6-propionoxyflavanone were grown from a THF (tetrahydrofurane) solution under ambient conditions.

### Refinement

Occupancy factors for C2, C2A, C11—C16 and C11A—C16A were refined. The C11A—C16A atoms were refined using ISOR restrain. All H atoms were placed at calculated positions. H atoms attached to carbons were constrained as riding atoms, with C–H set to 0.95 - 0.99 Å.  U_iso_(H) values were set to 1.2U_eq_ of the parent atom.

### Crystal data

**Table:**

C_18_H_16_O_4_	*F*(000) = 624
*M**_r_* = 296.31	*D*_x_ = 1.331 Mg m^−^^3^
Monoclinic, *P*2_1_/*n*	Mo *K*α radiation, λ = 0.71073 Å
Hall symbol: -P 2yn	Cell parameters from 2758 reflections
*a* = 7.863 (2) Å	θ = 2.9–36.8°
*b* = 17.876 (4) Å	µ = 0.09 mm^−^^1^
*c* = 10.731 (2) Å	*T* = 100 K
β = 101.28 (3)°	Plate, colorless
*V* = 1479.2 (6) Å^3^	0.32 × 0.15 × 0.09 mm
*Z* = 4	

### Data collection

**Table:**

Kuma KM4 CCD diffractometer	1906 reflections with *I* > 2σ(*I*)
Radiation source: fine-focus sealed tube	*R*_int_ = 0.126
graphite	θ_max_ = 33.0°, θ_min_ = 2.9°
ω scan	*h* = −9→12
23501 measured reflections	*k* = −27→27
5512 independent reflections	*l* = −16→16

### Refinement

**Table:**

Refinement on *F*^2^	Primary atom site location: structure-invariant direct methods
Least-squares matrix: full	Secondary atom site location: difference Fourier map
*R*[*F*^2^ > 2σ(*F*^2^)] = 0.063	Hydrogen site location: inferred from neighbouring sites
*wR*(*F*^2^) = 0.140	H-atom parameters constrained
*S* = 0.86	*w* = 1/[σ^2^(*F*_o_^2^) + (0.0501*P*)^2^] where *P* = (*F*_o_^2^ + 2*F*_c_^2^)/3
5512 reflections	(Δ/σ)_max_ < 0.001
263 parameters	Δρ_max_ = 0.26 e Å^−^^3^
186 restraints	Δρ_min_ = −0.20 e Å^−^^3^

### Special details

**Table:**

Geometry. All esds (except the esd in the dihedral angle between two l.s. planes) are estimated using the full covariance matrix. The cell esds are taken into account individually in the estimation of esds in distances, angles and torsion angles; correlations between esds in cell parameters are only used when they are defined by crystal symmetry. An approximate (isotropic) treatment of cell esds is used for estimating esds involving l.s. planes.
Refinement. Refinement of *F*^2^ against ALL reflections. The weighted *R*-factor wR and goodness of fit *S* are based on *F*^2^, conventional *R*-factors *R* are based on *F*, with *F* set to zero for negative *F*^2^. The threshold expression of *F*^2^ > σ(*F*^2^) is used only for calculating *R*-factors(gt) etc. and is not relevant to the choice of reflections for refinement. *R*-factors based on *F*^2^ are statistically about twice as large as those based on *F*, and *R*- factors based on ALL data will be even larger.

### Fractional atomic coordinates and isotropic or equivalent isotropic displacement parameters (Å^2^)

**Table:**

	*x*	*y*	*z*	*U*_iso_*/*U*_eq_	Occ. (<1)
O1	0.08199 (17)	0.09868 (7)	0.26522 (11)	0.0360 (3)	
C3	−0.2206 (2)	0.11679 (11)	0.28343 (16)	0.0333 (5)	
H3C	−0.2405	0.1702	0.2607	0.040*	0.191 (5)
H3D	−0.3312	0.0899	0.2539	0.040*	0.191 (5)
H3A	−0.3199	0.1516	0.2612	0.040*	0.809 (5)
H3B	−0.2583	0.0672	0.2469	0.040*	0.809 (5)
C2	−0.0717 (3)	0.14510 (17)	0.2248 (2)	0.0291 (7)	0.809 (5)
H2	−0.0437	0.1975	0.2544	0.035*	0.809 (5)
C2A	−0.0799 (12)	0.0847 (7)	0.2107 (8)	0.029 (3)	0.191 (5)
H2A	−0.0935	0.0290	0.2091	0.034*	0.191 (5)
O4	−0.27867 (17)	0.11284 (9)	0.49357 (12)	0.0510 (4)	
C4	−0.1720 (3)	0.11027 (12)	0.42566 (17)	0.0367 (5)	
C5	0.0761 (3)	0.09439 (11)	0.60752 (17)	0.0354 (5)	
H5	−0.0024	0.0986	0.6641	0.042*	
C6	0.2489 (2)	0.08326 (10)	0.65442 (16)	0.0325 (5)	
C7	0.3650 (3)	0.07588 (11)	0.57403 (18)	0.0378 (5)	
H7	0.4845	0.0680	0.6080	0.045*	
C8	0.3072 (2)	0.07993 (11)	0.44393 (17)	0.0375 (5)	
H8	0.3865	0.0740	0.3883	0.045*	
C9	0.1325 (2)	0.09266 (10)	0.39496 (16)	0.0300 (4)	
C10	0.0149 (2)	0.09958 (10)	0.47656 (16)	0.0310 (4)	
C11	−0.1170 (4)	0.1450 (3)	0.0831 (3)	0.0303 (7)	0.809 (5)
C12	−0.1411 (5)	0.0793 (2)	0.0138 (4)	0.0366 (9)	0.809 (5)
H12	−0.1219	0.0327	0.0570	0.044*	0.809 (5)
C13	−0.1934 (7)	0.0798 (3)	−0.1190 (4)	0.0397 (10)	0.809 (5)
H13	−0.2060	0.0342	−0.1652	0.048*	0.809 (5)
C14	−0.2265 (7)	0.1475 (3)	−0.1823 (5)	0.0382 (11)	0.809 (5)
H14	−0.2649	0.1483	−0.2719	0.046*	0.809 (5)
C15	−0.2034 (5)	0.2140 (2)	−0.1141 (3)	0.0417 (8)	0.809 (5)
H15	−0.2251	0.2605	−0.1572	0.050*	0.809 (5)
C16	−0.1478 (4)	0.2128 (2)	0.0186 (3)	0.0376 (7)	0.809 (5)
H16	−0.1311	0.2585	0.0646	0.045*	0.809 (5)
C11A	−0.119 (2)	0.1114 (11)	0.0698 (17)	0.034 (3)	0.191 (5)
C12A	−0.172 (2)	0.0636 (11)	−0.0273 (17)	0.037 (3)	0.191 (5)
H12A	−0.1752	0.0112	−0.0136	0.045*	0.191 (5)
C13A	−0.220 (3)	0.0935 (16)	−0.147 (2)	0.040 (3)	0.191 (5)
H13A	−0.2685	0.0616	−0.2153	0.048*	0.191 (5)
C14A	−0.201 (3)	0.1676 (14)	−0.170 (2)	0.035 (2)	0.191 (5)
H14A	−0.2292	0.1866	−0.2541	0.042*	0.191 (5)
C15A	−0.142 (2)	0.2133 (9)	−0.0714 (15)	0.038 (2)	0.191 (5)
H15A	−0.1262	0.2652	−0.0847	0.046*	0.191 (5)
C16A	−0.1042 (19)	0.1832 (10)	0.0494 (14)	0.033 (2)	0.191 (5)
H16A	−0.0662	0.2154	0.1197	0.040*	0.191 (5)
O17	0.30900 (16)	0.07682 (7)	0.78730 (11)	0.0366 (3)	
O18	0.28558 (19)	0.20177 (8)	0.80597 (12)	0.0481 (4)	
C18	0.3155 (2)	0.14193 (13)	0.85498 (18)	0.0368 (5)	
C19	0.3615 (3)	0.12719 (12)	0.99504 (17)	0.0429 (5)	
H19A	0.4743	0.1008	1.0143	0.051*	
H19B	0.2729	0.0937	1.0192	0.051*	
C20	0.3734 (3)	0.19745 (13)	1.07435 (19)	0.0525 (6)	
H20A	0.4030	0.1842	1.1646	0.079*	
H20B	0.2615	0.2234	1.0570	0.079*	
H20C	0.4632	0.2303	1.0529	0.079*	

### Atomic displacement parameters (Å^2^)

**Table:**

	*U*^11^	*U*^22^	*U*^33^	*U*^12^	*U*^13^	*U*^23^
O1	0.0336 (8)	0.0530 (9)	0.0217 (7)	0.0076 (6)	0.0058 (6)	0.0035 (6)
C3	0.0309 (10)	0.0463 (12)	0.0221 (9)	−0.0009 (9)	0.0037 (8)	0.0026 (8)
C2	0.0281 (13)	0.0357 (18)	0.0218 (11)	0.0042 (12)	0.0009 (9)	0.0039 (11)
C2A	0.029 (6)	0.044 (8)	0.015 (4)	−0.001 (5)	0.009 (4)	0.006 (4)
O4	0.0312 (8)	0.0941 (12)	0.0288 (7)	−0.0055 (8)	0.0086 (6)	0.0014 (8)
C4	0.0309 (10)	0.0531 (13)	0.0255 (10)	−0.0048 (10)	0.0045 (9)	−0.0011 (9)
C5	0.0328 (11)	0.0499 (14)	0.0244 (10)	−0.0013 (9)	0.0081 (8)	0.0012 (9)
C6	0.0368 (11)	0.0385 (12)	0.0220 (9)	0.0016 (9)	0.0050 (8)	0.0033 (8)
C7	0.0312 (11)	0.0504 (13)	0.0309 (10)	0.0068 (10)	0.0037 (8)	0.0064 (9)
C8	0.0321 (11)	0.0543 (14)	0.0264 (10)	0.0089 (10)	0.0062 (8)	0.0044 (9)
C9	0.0351 (11)	0.0333 (11)	0.0207 (9)	0.0024 (9)	0.0030 (8)	0.0024 (8)
C10	0.0297 (10)	0.0398 (12)	0.0235 (10)	−0.0008 (8)	0.0051 (8)	0.0019 (8)
C11	0.0287 (13)	0.039 (2)	0.0252 (14)	0.0019 (18)	0.0085 (10)	−0.0011 (15)
C12	0.0418 (19)	0.041 (2)	0.0261 (19)	0.0011 (16)	0.0045 (16)	0.0034 (16)
C13	0.042 (2)	0.050 (2)	0.027 (2)	0.0019 (17)	0.0054 (16)	0.0004 (16)
C14	0.036 (2)	0.057 (3)	0.0211 (15)	0.0063 (19)	0.0045 (13)	−0.0009 (18)
C15	0.049 (2)	0.0538 (18)	0.0220 (16)	0.0168 (16)	0.0050 (13)	0.0067 (15)
C16	0.0494 (18)	0.0398 (18)	0.0238 (15)	0.0082 (14)	0.0077 (12)	0.0030 (13)
C11A	0.038 (4)	0.036 (5)	0.028 (4)	0.003 (4)	0.004 (4)	−0.004 (4)
C12A	0.040 (4)	0.045 (4)	0.026 (5)	0.003 (4)	0.004 (4)	−0.004 (4)
C13A	0.038 (4)	0.052 (5)	0.029 (5)	0.006 (4)	0.005 (4)	0.004 (4)
C14A	0.037 (4)	0.047 (4)	0.023 (4)	0.006 (4)	0.011 (4)	0.005 (4)
C15A	0.043 (4)	0.041 (4)	0.028 (4)	0.008 (4)	0.001 (4)	0.003 (4)
C16A	0.041 (4)	0.032 (5)	0.025 (4)	0.012 (4)	0.003 (4)	−0.001 (4)
O17	0.0386 (8)	0.0457 (9)	0.0231 (7)	0.0039 (7)	−0.0001 (6)	0.0015 (6)
O18	0.0642 (10)	0.0454 (9)	0.0318 (8)	−0.0007 (8)	0.0023 (7)	0.0045 (7)
C18	0.0311 (11)	0.0506 (14)	0.0284 (10)	0.0003 (10)	0.0047 (8)	0.0015 (10)
C19	0.0454 (12)	0.0564 (14)	0.0252 (10)	0.0072 (11)	0.0027 (9)	0.0026 (9)
C20	0.0544 (14)	0.0722 (17)	0.0289 (11)	0.0118 (12)	0.0032 (10)	−0.0057 (11)

### Geometric parameters (Å, °)

**Table:**

O1—C2A	1.317 (10)	C12—C13	1.404 (5)
O1—C9	1.375 (2)	C12—H12	0.9500
O1—C2	1.460 (3)	C13—C14	1.386 (6)
C3—C4	1.504 (2)	C13—H13	0.9500
C3—C2	1.520 (3)	C14—C15	1.390 (6)
C3—C2A	1.581 (10)	C14—H14	0.9500
C3—H3C	0.9900	C15—C16	1.405 (4)
C3—H3D	0.9900	C15—H15	0.9500
C3—H3A	0.9899	C16—H16	0.9500
C3—H3B	0.9901	C11A—C16A	1.31 (2)
C2—C11	1.492 (4)	C11A—C12A	1.35 (2)
C2—H2	1.0000	C12A—C13A	1.37 (2)
C2A—C11A	1.558 (19)	C12A—H12A	0.9500
C2A—H3B	1.5579	C13A—C14A	1.36 (3)
C2A—H2A	1.0000	C13A—H13A	0.9500
O4—C4	1.215 (2)	C14A—C15A	1.35 (3)
C4—C10	1.476 (3)	C14A—H14A	0.9500
C5—C6	1.368 (3)	C15A—C16A	1.381 (19)
C5—C10	1.397 (2)	C15A—H15A	0.9500
C5—H5	0.9500	C16A—H16A	0.9500
C6—C7	1.379 (3)	O17—C18	1.367 (2)
C6—O17	1.416 (2)	O18—C18	1.195 (2)
C7—C8	1.383 (3)	C18—C19	1.499 (3)
C7—H7	0.9500	C19—C20	1.510 (3)
C8—C9	1.390 (3)	C19—H19A	0.9900
C8—H8	0.9500	C19—H19B	0.9900
C9—C10	1.398 (2)	C20—H20A	0.9800
C11—C12	1.383 (4)	C20—H20B	0.9800
C11—C16	1.394 (4)	C20—H20C	0.9800
			
C2A—O1—C9	119.8 (4)	C8—C9—C10	120.23 (16)
C2A—O1—C2	45.9 (5)	C5—C10—C9	118.98 (18)
C9—O1—C2	113.80 (15)	C5—C10—C4	120.20 (17)
C4—C3—C2	112.49 (16)	C9—C10—C4	120.80 (16)
C4—C3—C2A	114.1 (4)	C12—C11—C16	118.7 (3)
C2—C3—C2A	41.1 (4)	C12—C11—C2	122.0 (4)
C4—C3—H3C	108.7	C16—C11—C2	119.2 (3)
C2—C3—H3C	71.0	C11—C12—C13	121.4 (4)
C2A—C3—H3C	108.7	C11—C12—H12	119.3
C4—C3—H3D	108.7	C13—C12—H12	119.3
C2—C3—H3D	136.7	C14—C13—C12	119.6 (5)
C2A—C3—H3D	108.7	C14—C13—H13	120.2
H3C—C3—H3D	107.6	C12—C13—H13	120.2
C4—C3—H3A	109.0	C13—C14—C15	119.7 (5)
C2—C3—H3A	109.0	C13—C14—H14	120.1
C2A—C3—H3A	134.8	C15—C14—H14	120.1
H3C—C3—H3A	41.9	C14—C15—C16	120.2 (4)
H3D—C3—H3A	68.1	C14—C15—H15	119.9
C4—C3—H3B	109.3	C16—C15—H15	119.9
C2—C3—H3B	109.2	C11—C16—C15	120.4 (3)
C2A—C3—H3B	70.3	C11—C16—H16	119.8
H3C—C3—H3B	138.1	C15—C16—H16	119.8
H3D—C3—H3B	42.5	C16A—C11A—C12A	121.0 (17)
H3A—C3—H3B	107.8	C16A—C11A—C2A	117.1 (16)
O1—C2—C11	108.7 (2)	C12A—C11A—C2A	121.9 (17)
O1—C2—C3	110.33 (18)	C11A—C12A—C13A	118 (2)
C11—C2—C3	111.8 (2)	C11A—C12A—H12A	121.2
O1—C2—H2	108.6	C13A—C12A—H12A	121.2
C11—C2—H2	108.6	C14A—C13A—C12A	122 (2)
C3—C2—H2	108.6	C14A—C13A—H13A	119.0
O1—C2A—C11A	111.4 (9)	C12A—C13A—H13A	119.0
O1—C2A—C3	114.9 (7)	C15A—C14A—C13A	119 (2)
C11A—C2A—C3	109.9 (9)	C15A—C14A—H14A	120.6
O1—C2A—H3B	140.0	C13A—C14A—H14A	120.6
C11A—C2A—H3B	106.5	C14A—C15A—C16A	118.6 (16)
C3—C2A—H3B	36.8	C14A—C15A—H15A	120.7
O1—C2A—H2A	106.7	C16A—C15A—H15A	120.7
C11A—C2A—H2A	106.7	C11A—C16A—C15A	121.9 (14)
C3—C2A—H2A	106.7	C11A—C16A—H16A	119.0
H3B—C2A—H2A	73.0	C15A—C16A—H16A	119.0
O4—C4—C10	122.48 (16)	C18—O17—C6	115.85 (15)
O4—C4—C3	122.51 (18)	O18—C18—O17	122.98 (18)
C10—C4—C3	115.01 (16)	O18—C18—C19	125.9 (2)
C6—C5—C10	120.12 (18)	O17—C18—C19	111.08 (18)
C6—C5—H5	119.9	C18—C19—C20	113.28 (19)
C10—C5—H5	119.9	C18—C19—H19A	108.9
C5—C6—C7	120.98 (17)	C20—C19—H19A	108.9
C5—C6—O17	119.59 (17)	C18—C19—H19B	108.9
C7—C6—O17	119.39 (17)	C20—C19—H19B	108.9
C6—C7—C8	119.97 (19)	H19A—C19—H19B	107.7
C6—C7—H7	120.0	C19—C20—H20A	109.5
C8—C7—H7	120.0	C19—C20—H20B	109.5
C7—C8—C9	119.70 (18)	H20A—C20—H20B	109.5
C7—C8—H8	120.1	C19—C20—H20C	109.5
C9—C8—H8	120.1	H20A—C20—H20C	109.5
O1—C9—C8	117.62 (17)	H20B—C20—H20C	109.5
O1—C9—C10	122.15 (17)		
			
C2A—O1—C2—C11	−68.7 (5)	C8—C9—C10—C4	−177.69 (18)
C9—O1—C2—C11	−177.9 (2)	O4—C4—C10—C5	−2.1 (3)
C2A—O1—C2—C3	54.2 (5)	C3—C4—C10—C5	178.64 (18)
C9—O1—C2—C3	−55.0 (2)	O4—C4—C10—C9	176.33 (19)
C4—C3—C2—O1	53.9 (3)	C3—C4—C10—C9	−2.9 (3)
C2A—C3—C2—O1	−47.5 (5)	O1—C2—C11—C12	53.9 (4)
C4—C3—C2—C11	175.0 (2)	C3—C2—C11—C12	−68.2 (4)
C2A—C3—C2—C11	73.6 (5)	O1—C2—C11—C16	−130.7 (3)
C9—O1—C2A—C11A	167.4 (8)	C3—C2—C11—C16	107.3 (3)
C2—O1—C2A—C11A	72.1 (9)	C16—C11—C12—C13	0.8 (5)
C9—O1—C2A—C3	41.6 (10)	C2—C11—C12—C13	176.3 (4)
C2—O1—C2A—C3	−53.7 (7)	C11—C12—C13—C14	−1.9 (7)
C4—C3—C2A—O1	−39.4 (10)	C12—C13—C14—C15	1.7 (8)
C2—C3—C2A—O1	57.7 (7)	C13—C14—C15—C16	−0.5 (7)
C4—C3—C2A—C11A	−165.9 (8)	C12—C11—C16—C15	0.4 (5)
C2—C3—C2A—C11A	−68.8 (9)	C2—C11—C16—C15	−175.2 (3)
C2—C3—C4—O4	155.4 (2)	C14—C15—C16—C11	−0.6 (5)
C2A—C3—C4—O4	−159.7 (5)	O1—C2A—C11A—C16A	−61.9 (18)
C2—C3—C4—C10	−25.4 (3)	C3—C2A—C11A—C16A	66.6 (17)
C2A—C3—C4—C10	19.6 (6)	O1—C2A—C11A—C12A	119.9 (17)
C10—C5—C6—C7	−1.0 (3)	C3—C2A—C11A—C12A	−111.6 (17)
C10—C5—C6—O17	−178.93 (17)	C16A—C11A—C12A—C13A	−5(3)
C5—C6—C7—C8	0.2 (3)	C2A—C11A—C12A—C13A	173.6 (17)
O17—C6—C7—C8	178.17 (18)	C11A—C12A—C13A—C14A	6(4)
C6—C7—C8—C9	1.0 (3)	C12A—C13A—C14A—C15A	−4(4)
C2A—O1—C9—C8	156.3 (7)	C13A—C14A—C15A—C16A	−1(3)
C2—O1—C9—C8	−152.26 (19)	C12A—C11A—C16A—C15A	0(3)
C2A—O1—C9—C10	−24.2 (7)	C2A—C11A—C16A—C15A	−177.7 (12)
C2—O1—C9—C10	27.2 (3)	C14A—C15A—C16A—C11A	2(3)
C7—C8—C9—O1	177.93 (18)	C5—C6—O17—C18	−74.2 (2)
C7—C8—C9—C10	−1.5 (3)	C7—C6—O17—C18	107.8 (2)
C6—C5—C10—C9	0.5 (3)	C6—O17—C18—O18	−5.5 (3)
C6—C5—C10—C4	178.93 (18)	C6—O17—C18—C19	173.63 (16)
O1—C9—C10—C5	−178.66 (17)	O18—C18—C19—C20	−1.8 (3)
C8—C9—C10—C5	0.8 (3)	O17—C18—C19—C20	179.05 (17)
O1—C9—C10—C4	2.9 (3)		

### Hydrogen-bond geometry (Å, °)

**Table:**

*D*—H···*A*	*D*—H	H···*A*	*D*···*A*	*D*—H···*A*
C2—H2···O18^i^	1.00	2.37	3.145 (3)	133

Symmetry codes: (i) *x*−1/2, −*y*+1/2, *z*−1/2.
